# Expert consensus on oral management in autoimmune bullous diseases, erythema multiforme and SJS/TEN

**DOI:** 10.1111/jdv.70578

**Published:** 2026-07-22

**Authors:** Shalini Nayee, Molly Harte, Saskia Ingen‐Housz‐Oro, Milad Ameri, Sarah Walsh, Pascal Joly, Michael Ardern‐Jones, Vlaho Brailo, Marie‐Charlotte Brueggen, Christopher Bunker, Barbara Carey, Marco Carrozzo, Ser‐Ling Chua, Daniel Creamer, Donna Culton, Konstantina Delli, Marcio Diniz‐Freitas, Roni Dodiuk‐Gad, Sophie‐Myriam Dridi, Anne‐Laure Ejeil, Lars French, Krisztian Gaspar, Jon Higham, Barbara Horvarth, Esther Hullah, Sabine Jurge, Roddy McMillan, Helen McParland, Dedee Murrell, Martyn Ormond, Ana Poveda‐Gallego, Mahtab Samimi, Enno Schmidt, Martina Shephard, Tom Sollecito, Jennifer Taylor, Soner Uzun, Skaidra Valiukeviciene, Amanda Willis, Jane Setterfield

**Affiliations:** ^1^ Department of Oral Medicine, Guy's Hospital Guy's and St Thomas' NHS Foundation Trust London UK; ^2^ Dermatology Department, AP‐HP Henri Mondor hospital Crétil France; ^3^ Reference center for toxic bullous dermatoses and severe drug reactions TOXIBUL Créteil France; ^4^ AIBD‐ToxiTEN group of the ERN‐skin Univ Paris Est Créteil, EpiDermE Créteil France; ^5^ Department of Dermatology University Hospital of Zurich Zurich Switzerland; ^6^ Department of Dermatology King's College Hospital London UK; ^7^ Department of Dermatology Rouen University Hospital, INSERM U1234, Normandie University Rouen France; ^8^ Department of Dermatology University Hospitals Southampton NHS Foundation Trust UK; ^9^ University of Zagreb School of Dental Medicine University Clinical Hospital Centre Zagreb Croatia; ^10^ Faculty of Medicine University of Zurich Zurich Switzerland; ^11^ Department of Dermatology Medical University of Vienna Vienna Austria; ^12^ Department of Dermatology University College London Hospitals London UK; ^13^ Department of Head & Neck Surgical Oncology Guy's Hospital London UK; ^14^ Department of Oral Medicine, School of Dental Sciences University of Newcastle upon Tyne UK; ^15^ Department of Dermatology University Hospitals Birmingham NHS Foundation Trust Birmingham UK; ^16^ Department of Dermatology University of North Carolina Chapel Hill North Carolina USA; ^17^ Department of Oral and Maxillofacial Surgery University of Groningen, University Medical Centre Groningen Groningen the Netherlands; ^18^ Center for Dentistry and Oral Hygiene University Medical Center Groningen and University of Groningen Groningen the Netherlands; ^19^ Special Care Dentistry Unit, School of Medicine and Dentistry University of Santiago de Compostela Santiago de Compostela Spain; ^20^ Medical‐Surgical Dentistry Research Group (OMEQUI) Health Research Institute of Santiago de Compostela (IDIS), University of Santiago de Compostela (USC) Santiago de Compostela Spain; ^21^ Department of Dermatology Emek Medical Center Afula Israel; ^22^ Department of Periodontology Fondation Université Côte d'Azur, Nice Provence‐Alpes‐Côte d'Azur France; ^23^ Department of Odontology Bretonneau Hospital AP‐HP Paris France; ^24^ Faculty of Odontology, Department of Oral Surgery Paris Cité University Montrouge France; ^25^ Inserm UMR1333 Oral Heath Montrouge France; ^26^ Department of Dermatology and Allergy University Hospital, LMU Munich Munich Germany; ^27^ Division of Dermatological Allergology, Faculty of Medicine University of Debrecen Debrecen Hungary; ^28^ Department of Dermatology, Faculty of Medicine University of Debrecen Debrecen Hungary; ^29^ Department of Oral Medicine Birmingham Dental Hospital and School of Dentistry Birmingham UK; ^30^ Department of Dermatology University Medical Centre Groningen University of Groningen Groningen The Netherlands; ^31^ Department of Oral Medicine Sheffield Teaching Hospitals NHS Foundation Trust and University of Sheffield Sheffield UK; ^32^ Department of Oral Medicine & Facial Pain Royal National ENT and Eastman Dental Hospitals, University College Hospitals, University College London Hospitals NHS Foundation Trust UK; ^33^ Department of Dermatology, St George Hospital Clinical School University of NSW Sydney Australia; ^34^ Dermatology Department University Hospital of Tours France; ^35^ Lübeck Institute of Experimental Dermatology University of Lübeck Lübeck Germany; ^36^ Department of Dermatology Universitätsklinikum Schleswig‐Holstein Lübeck Germany; ^37^ School of Dental Medicine University of Pennsylvania USA; ^38^ Oral Medicine, University of Glasgow Glasgow Dental Hospital and School Glasgow UK; ^39^ Department of Dermatology, Faculty of Medicine Akdeniz University Antalya Turkey; ^40^ Department of Skin and Venereal Diseases The Lithuanian University of Health Sciences Kaunas Lithuania; ^41^ Department of Oral Medicine Queens University Belfast, Belfast Health and Social Care Trust Belfast UK; ^42^ St John's Institute of Dermatology Guy's Hospital London UK; ^43^ Centre for Host Microbiome Interactions (CHMI), Faculty of Dentistry, Oral & Craniofacial Sciences King's College London London UK

**Keywords:** autoimmune diseases, consensus development, Delphi technique, epidermal necrolysis, epidermolysis bullosa, erythema multiforme, mouth mucosa, Oral hygiene, Oral manifestations, Oral medicine, mucous membrane pemphigoid, pemphigus, skin diseases, Stevens‐Johnson syndrome, Stevens‐Johnson syndrome toxic epidermal necrolysis Spectrum, toxic, vesiculobullous

## Abstract

**Background:**

Autoimmune bullous diseases (AIBD), Stevens‐Johnson syndrome and toxic epidermal necrolysis (SJS‐TEN) and erythema multiforme (EM) often present with clinically similar features in the oral mucosa: classically widespread mucosal erosions or ulceration. Despite this, there are no formalized guidelines for the specific management of the oral manifestations of these conditions.

**Objectives:**

We sought to establish an consensus on the management of oral involvement of AIBD, SJS‐TEN and EM using the Delphi method.

**Methods:**

Participants were sent a survey comprising 62 statements organized into 6 categories: general oral and dental recommendations; disease severity scoring; topical therapies for active oral involvement in AIBD; systemic therapies for active oral involvement in AIBD; additional considerations for inpatient care of AIBD, EM, SJS‐TEN; specific considerations for EM and SJS‐TEN. Participants rated the level of appropriateness of each statement. Results were analysed using the RAND/UCLA Appropriateness Method.

**Results:**

34 experts, primarily Oral Medicine clinicians and Dermatologists, completed the survey. Consensus was achieved for 58 (93.5%) statements after a single round. General recommendations included rigorous oral hygiene and regular dental/periodontal care. Disease‐specific severity scoring was recommended to guide treatment and monitor response. For active oral AIBD, experts recommended moderate‐to‐super potent topical corticosteroids, guided by severity and site of oral disease. For inpatients, supported oral hygiene, regular topical anaesthetics and nutritional support for impaired intake were recommended. For acute EM and SJS‐TEN, recommendations included regular lip care with gentle debridement of haemorrhagic crusts and application of soft paraffin.

**Conclusions:**

This Delphi sets out a broad framework for the management of oral involvement in AIBD, SJS‐TEN and EM, to be adapted by a multidisciplinary team, for both acute and long‐term management of these conditions. It may help to standardize the management of oral manifestations of these conditions in future multicentre studies.

## Graphical abstract

This international Delphi study achieved expert consensus on 58 statements guiding the management of oral involvement in autoimmune bullous diseases, erythema multiforme and SJS‐TEN. The recommendations emphasize multidisciplinary care, oral hygiene and tailored topical, systemic and inpatient oral management.

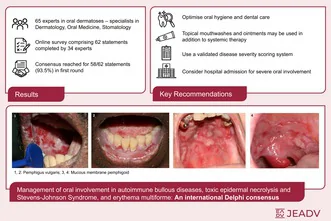

**Why was the study undertaken?**

Oral involvement is a common finding in autoimmune bullous disease (AIBD), erythema multiforme (EM) and Stevens‐Johnson syndrome and toxic epidermal necrolysis (SJS‐TEN). The oral manifestations of these conditions are distinct from the cutaneous disease and can require specific management. Despite this, no current evidence‐based guidelines or formalized recommendations exist. This study aims to establish a consensus on the oral management of AIBD, EM and SJS‐TEN using the Delphi method.

**What does this study add?**

This study provides 58 consensus‐based recommendations for the oral management of AIBD, EM and SJS‐TEN. Prioritization of oral hygiene and regular periodontal care was recommended. The study presents recommendations to guide the use of topical corticosteroid preparations in these diseases, based on site and severity of disease. It provides a series of recommendations for inpatient care, covering topical anaesthetics, nutritional support and lip care. It outlines recommendations regarding the identification and management of infective triggers for EM.

**What are the implications of this study for disease understanding and/or clinical care?**

This study provides an expert consensus on the specific management of oral mucosal involvement in AIBD, EM and SJS‐TEN, providing recommendations for clinicians involved in the management of these conditions. It may help to standardize management of oral manifestations of AIBD, EM and SJS‐TEN, ensuring patients receive tailored and specific clinical care appropriate to their individual presentation. It provides a foundation for future multicentre clinical trials focused on management and outcomes of oral mucosal disease in these conditions.

## INTRODUCTION

Autoimmune bullous diseases (AIBDs) are a group of chronic conditions characterized by auto‐antibody mediated targeting of structural proteins within skin and mucous membranes.^1^ Oral involvement is a common feature of AIBD, especially in pemphigus vulgaris, mucous membrane pemphigoid and epidermolysis bullosa acquisita and is often the first site of disease activity.^2^, ^3^ Oral manifestations may include persistent oral erosions (e.g. pemphigus vulgaris), ulcers (e.g. mucous membrane pemphigoid) and desquamative gingivitis (Figure 1), and pain associated with these lesions may significantly impact on speech, nutrition and quality of life.^3^, ^4^

**FIGURE 1:**
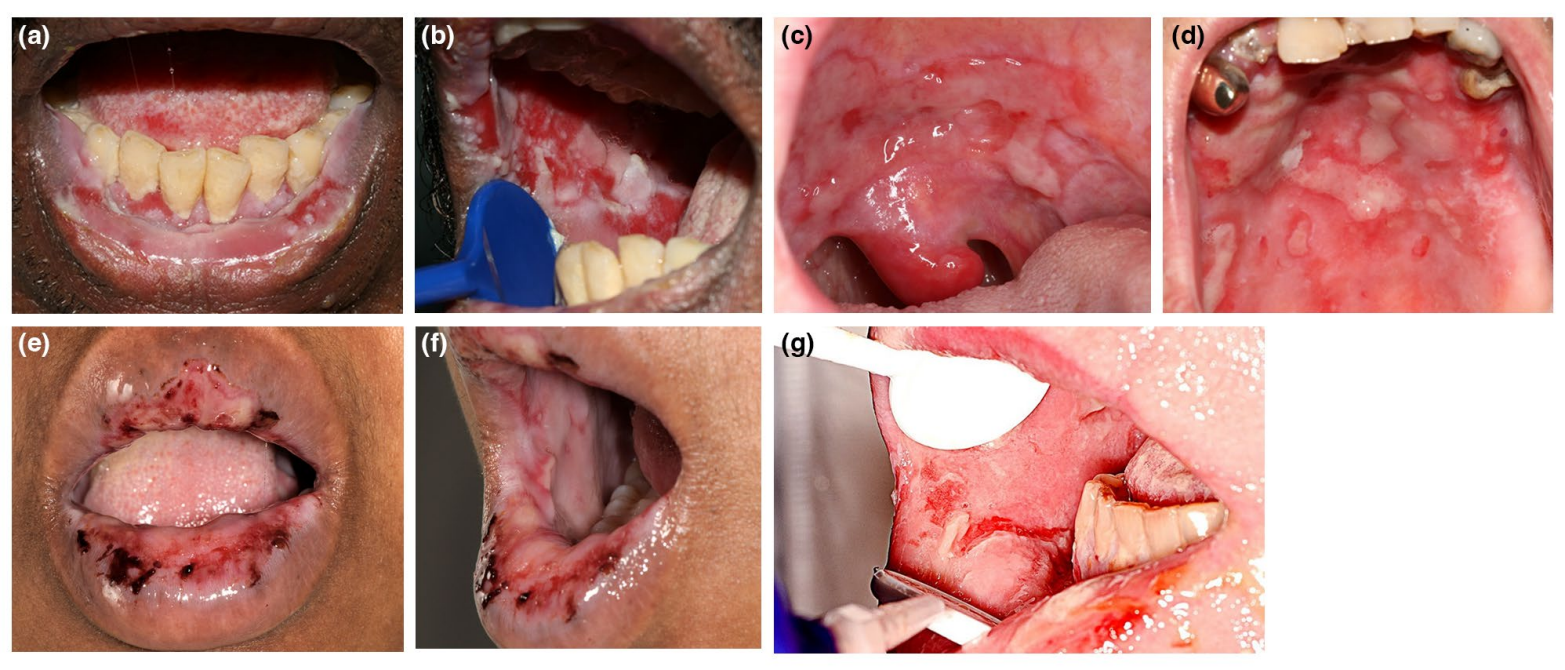
Clinical images of oral manifestations of Pemphigus vulgaris (A, B), Mucous Membrane Pemphigoid (C, D), EM (E, F) and SJS‐TEN (G).

Erythema Multiforme (EM) is an acute and often recurrent mucocutaneous presentation that may be triggered by infection, most notably herpes simplex virus (HSV).^5^, ^6^ The oral presentation of EM is highly variable but may include widespread oral ulceration and haemorrhagic crusting of the lips.^6^, ^7^ Whilst EM is often a self‐limiting presentation, the extent of oral involvement can be severe and debilitating, sometimes necessitating hospital admission to support adequate fluid and nutritional intake.^8^, ^9^

Stevens‐Johnson syndrome and toxic epidermal necrolysis (SJS‐TEN) is a rare spectrum of potentially life‐threatening delayed hypersensitivity reactions, most commonly induced by medication.^5^, ^10^ Whatever the extent of skin lesions (with SJS affecting <10% body surface area (BSA), SJS‐TEN between 10 and 30% and TEN >30% BSA),^11^ oral lesions in SJS‐TEN are often extensive and painful, with widespread ulceration affecting the oral cavity and oropharynx and may carry a risk of long‐term sequelae including the formation of fibrotic adhesions.^12^

The oral manifestations of AIBD, EM and SJS‐TEN are clinically similar and share the same clinical consequences in terms of impact on food intake and quality of life. Beside the specific treatment of the underlying condition, supportive care is required to limit the consequences of extensive oral lesions. Furthermore, these conditions are often slow to respond to treatment and may follow a different clinical course to the cutaneous disease.^4^ Tailored management approaches for the oral mucosal manifestations of these conditions, differing from the standard management strategies employed for the cutaneous disease, are often required. There are currently no formalized guidelines or recommendations for management of oral mucosal disease in this group of conditions.

The aim of this Delphi study was to define an expert consensus on the management of the oral manifestations of AIBD, EM and SJS‐TEN, improving supportive care in these conditions.

## METHODS

### Steering committee, literature review and statement development

A steering committee (J.S., S.IHO., S.W., S.N., M.H.) consisting of Dermatology and Oral Medicine clinicians from the AIBD‐ToxiTEN (SJS‐TEN and EM) group of the ERN‐skin, was established. A literature review was performed in PubMed with combinations of the following search terms: *pemphigus vulgaris, mucous membrane pemphigoid, epidermolysis bullosa acquisita, dermatitis herpetiformis, paraneoplastic pemphigus, paraneoplastic autoimmune multiorgan syndrome, erythema multiforme, Stevens‐Johnson syndrome, toxic epidermal necrolysis, oral, treatment, management, oral scoring*. Meta‐analyses, systematic reviews, clinical trials, original reviews and case reports/series published in English between 2015 and 2024 and considered of interest for this Delphi exercise were included.

The steering committee agreed on 6 categories to be addressed by the Delphi^1^: General recommendations, oral hygiene and dental care,^2^ Disease severity scoring,^3^ Topical therapies for active oral involvement in AIBD,^4^ Systemic therapies for active oral involvement in AIBD,^5^ Additional considerations for inpatient care of AIBD, EM, SJS‐TEN,^6^ Specific considerations for EM and SJS‐TEN. Following review of the literature, the steering committee drafted and developed initial statements. Sixty‐two statements were agreed upon by the steering committee.

The Delphi exercise took place from December 2024 to February 2025. This study was not subject to ethical approval as it did not involve patient data or samples.

### Panel selection

An international multidisciplinary panel of experts in the fields of AIBD, SJS‐TEN and EM was invited to participate in this Delphi exercise. Experts were identified through their participation in expert dermatology networks or involvement in specialist networks within Oral Medicine. Oral Medicine is a dental specialty, where clinicians may have an additional medical qualification. However, Oral Medicine does not exist as a recognized specialty in many European countries and therefore clinicians working in other dental specialties (such as Periodontology and Oral Surgery) may have a special interest in Oral Medicine. A total of 65 experts, from across a range of relevant medical and dental specialties with daily experience in managing oral mucosal disease, working in Europe, North America, Australia and Asia, were invited to participate in the Delphi exercise via email. Specifically, the experts comprised 29 Oral Medicine specialists, 27 Dermatologists, 5 Oral Surgeons, 2 Periodontologists, 1 Special Care Dentist and 1 Dermatology clinical nurse specialist (Figure 2a).

**FIGURE 2:**
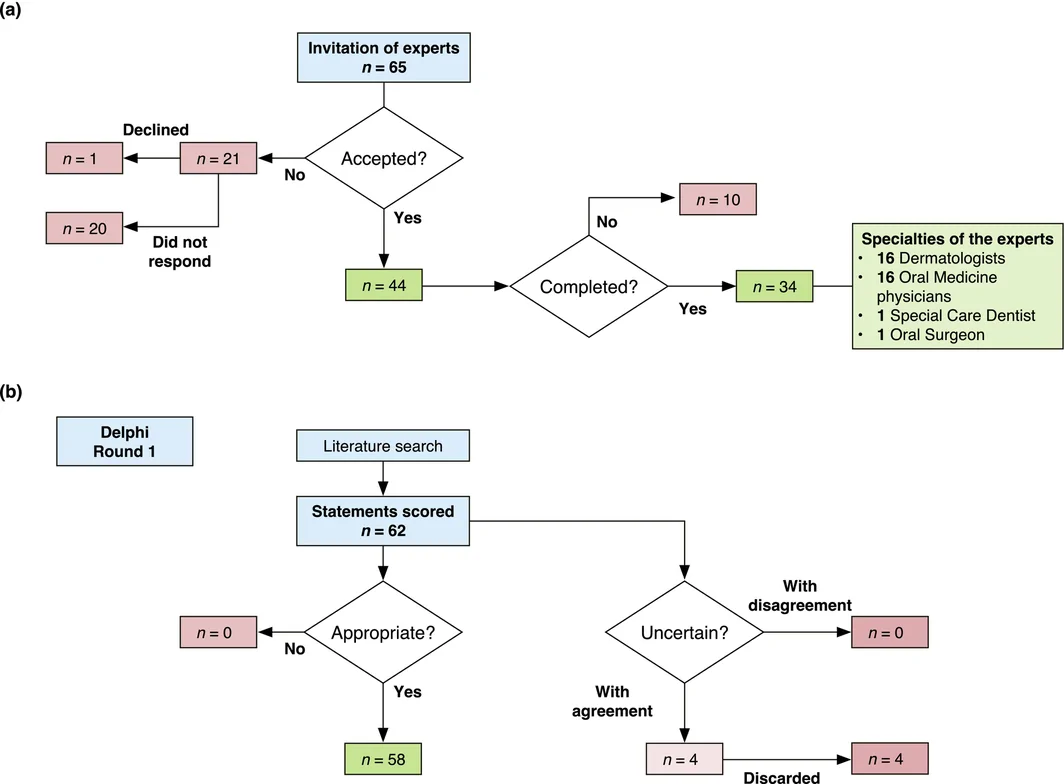
(a) Flowchart of the expert panel selection. (b) Flowchart of the first round Delphi exercise. 62 statements were scored with 58 agreed appropriate and the remaining 4 discarded. A second round of voting was not required.

### First round

In the first round (Figure 2b), participants received an online survey consisting of 62 statements regarding aspects of oral care in AIBD, EM and SJS‐TEN. The survey was sent via email using SurveyMonkey, an online tool (https://www.surveymonkey.co.uk), with a deadline for entering responses. Within this period, two personalized reminders were sent out to experts who had not yet evaluated the Delphi statements. Statements were organized into the six categories described above. Participants were asked to evaluate their level of agreement with each statement using a scale from 1 (strongly disagree) to 9 (strongly agree). Participants were asked not to rank statements they felt to be outside of their area of expertise. Participants had the opportunity to submit comments along with their evaluation for all statements. The steering committee (J.S., S.IHO., S.W., S.N., M.H.) did not respond to the survey.

### Statistical analysis

The survey responses were analysed using the RAND/UCLA Appropriateness method. For each statement, median appropriateness scores, interpercentile range (IPR), symmetry‐adjusted interpercentile range (IPRas) and the disagreement index (DI; ratio of IPR to IPRas) were calculated.^4^, ^13^ The median appropriateness score categorized as inappropriate (median 1.0–3.4), uncertain (median 3.5–6.9) or appropriate (median 7.0–9.0). Consensus was evaluated using the disagreement index (DI), with consensus defined as DI below 1 and lack of consensus as DI of 1 or higher.

## RESULTS

### Participants and Delphi exercise

Of the 65 experts invited to participate in the Delphi, one declined to participate due to insufficient subject expertise and 20 did not respond. Forty‐four experts agreed to participate, and in the first round, 34 experts responded to the survey (response rate: 52.3% of the 65 invited experts) (Figure 2a). Of the 34 experts, 19 were female and 15 were male. The experts were from 13 countries (Table S1).

### First round results

After a single round of the Delphi, consensus (defined as DI <1) was reached for 58 of 62 (93.5%) statements (Figure 2b). Four statements were classed as uncertain with agreement. These related to topical therapies for active oral involvement in AIBD, specifically: (1) use of hydrogen peroxide mouthwash; (2) duration of topical corticosteroids in AIBD; (3) use of antiseptics more than twice a day, in addition to (4) the role of HSV serology in selecting prophylactic antiviral treatment for EM. Following review by the steering committee, these 4 statements were discarded.

In view of the high level of consensus for the majority of statements, it was agreed that no further statement development or rounds of the Delphi were required.

All statements with the corresponding DIs and medians are presented in Table S2.

## DISCUSSION

The goal of this Delphi was to provide general recommendations for the management of oral involvement in AIBD, SJS‐TEN and EM, conditions included in the spectrum of diseases managed by the AIBD‐ToxiTEN group of the ERN‐skin and sharing the common challenge of extensive oral lesions with impact on quality of life. Fifty‐eight statements achieved consensus amongst the panel of experts, comprising Dermatologists and dental specialists (primarily Oral Medicine) from 13 countries.

General principles of care agreed upon by the participants include the importance of multidisciplinary working for patients with multi‐site involvement. It was acknowledged that patients with isolated oral involvement may be managed by expert oral medicine specialists/stomatologists alone. However, the scope of practice of oral medicine specialists/stomatologists will vary between healthcare systems and this statement must therefore be considered in the context of the scope of practice, conventions and regulatory environment of the country in which clinicians are practising. It was agreed that principles of oral hygiene and dental care should be prioritized and emphasized to patients (Table S2). The experts agreed that poor oral hygiene may exacerbate oral lesions and that oral lesions may take longer to respond to topical and systemic therapies than cutaneous sites of disease, as previously discussed in the literature.^4^, ^14^ Recommendations included smoking cessation, where applicable; seeking regular professional periodontal care every 3–6 months, with consideration of treatment under local anaesthetic; and deferring complex treatment such as dental implants until their oral disease has stabilized and oral hygiene has been optimized. Clinicians should also be aware of the potential for superinfection and arrange appropriate investigations and treatment. Specific oral hygiene recommendations included use of sodium‐lauryl‐sulphate (SLS)‐free toothpastes, considering use of a soft toothbrush and use of an antiseptic mouthwash once daily. Emphasis on oral care may be particularly relevant for patients with MMP, where the gingiva is the most frequent site of oral disease involvement. A point of uncertain agreement amongst participants was the use of hydrogen peroxide 1.5% mouthwash to aid oral hygiene, with concerns over mucosal irritation, lack of product availability and experts being unfamiliar with its use. In recognition of the potential impact of oral involvement, it was agreed that patients with impaired oral intake should receive advice from a dietician. However, this may not be feasible in all healthcare systems.

Severity scoring systems such as the Pemphigus Disease Area Index (PDAI), Mucous Membrane Pemphigoid Disease Area Index (MMPDAI), Autoimmune Bullous Skin Intensity and Severity Score (ABSIS) and oral disease severity score (ODSS) are used to record disease severity and guide treatment in AIBD.^3^, ^15^ There was consensus that disease‐specific severity scoring tools should be used in the assessment of oral mucosal disease to accurately capture oral mucosal disease activity, guide treatment decisions regarding topical and/or systemic therapy and allow assessment of treatment response. In free text comments, however, many experts commented on the practical challenges of calculating and recording specific disease severity scores under the time pressure of the clinical setting. Of note, there is no published severity score for oral lesions of SJS‐TEN and EM, where extension is not correlated to the skin BSA involved.

Topical therapies form an important role in the management of oral involvement in AIBD, with topical corticosteroids considered to be the primary treatment for oral involvement in MMP.^14^ Participants agreed that moderate‐potency topical corticosteroid mouthwash preparations should be used for mild oral disease, with addition of super‐potent corticosteroid ointments for more severe disease, localized gingival disease and as a potential maintenance treatment. Corticosteroid mouthwashes should be used at a separate time to antiseptic mouthwashes. Use of moderate‐potency corticosteroids or topical tacrolimus for external lip involvement once to twice daily until lesion healing was also agreed upon. Super‐potent corticosteroid ointments should not routinely be used for external lip involvement in AIBD. In many settings, moderate‐potency corticosteroid mouthwashes are used as a long‐term treatment and super‐potent corticosteroid preparations are used on a short‐term basis.^14^ However, there was uncertain agreement amongst participants on the duration of use of super‐potent topical corticosteroids. One potential consequence of regular topical corticosteroid use is oral candidiasis and experts agreed that combining high/super‐potent topical corticosteroid therapy with an antifungal agent should be considered. However, it is noted that this widely used clinical regimen may not be supported by local or national antimicrobial stewardship policies. Topical tacrolimus and intralesional corticosteroid injections were also agreed as potential adjunctive treatments. This lack of consensus may reflect that many topical preparations used in the oral cavity are prescribed ‘off‐label’. This is further exacerbated by the lack of trials focused on topical therapeutic use for oral involvement in AIBD, EM and SJS‐TEN.

Regarding the role of systemic therapy for oral involvement in AIBD, there was expert agreement that systemic therapy must be considered for moderate‐to‐severe oral lesions of any AIBD. The choice of systemic therapy must be guided by the specific AIBD diagnosis, site of involvement and the most up‐to‐date expert group guidelines, with use of the minimal effective dose to limit side effects.^3^, ^11^, ^14^, ^15^ There was expert agreement that topical therapies and oral hygiene recommendations should be continued alongside systemic therapy.

Where patients are admitted as an inpatient for management of EM, SJS‐TEN or AIBD, experts agreed that oral hygiene should be supported by a trained health care professional, where available. The consensus was that antiseptic mouthwashes, such as chlorhexidine 0.2%, should be used twice daily for inpatients. There was uncertain agreement about more frequent regimens (e.g. 4–6 times daily as performed by some teams, especially for SJS‐TEN). Concerning the use of chlorhexidine mouthwash, several participants specified the need for this to be alcohol‐free. It was agreed that the mouthwash may be applied with a sponge where patients are unable to rinse or expectorate. However, comments on this statement included the importance of reviewing local policies, as intra‐oral use of mouth sponges is restricted/banned in some health systems, due to the potential choking risk. Clinicians should therefore follow their local hospital policies. The importance of regular topical oral analgesia was also agreed upon. For patients with external lip involvement in the inpatient setting, regular lip care with yellow soft paraffin was agreed upon, with soaking and debridement of the lips with damp gauze to facilitate placement of ointments.

Regarding specific care for patients with EM or SJS‐TEN, it was agreed that hospital admission should be considered for patients with severe oral involvement of EM, with management for SJS‐TEN guided by the most up‐to‐date guidelines. There was also agreement that enteral nutrition should be provided where there is extensive oral involvement. Topical and local care comprising sweeping of the oral mucosa (with a sponge or as per local policies), potential use of topical corticosteroids ointments and mouthwashes for lip/oral involvement and topical tranexamic acid to manage haemorrhagic lips also achieved agreement. Potential prescription of short courses of systemic corticosteroids at the time of diagnosis of acute EM was also agreed upon. Screening and management of infectious triggers (such as *Herpes Simplex Virus* or *Mycoplasma pneumoniae* if associated with associated pulmonary symptoms) was also supported. Following the acute phase of disease, there was agreement that patients require follow‐up visits to assess complete healing of the oral mucosa. For recurrent HSV‐related EM (defined as 2 or more episodes in one year), there was agreement on the use of prophylactic antiviral treatment for 6–12 months. However, there was uncertain agreement about whether HSV serology should guide prophylactic treatment in recurrent EM, reflecting the varied practice observed in the literature.^16^ Comments regarding this included the lack of access to HSV serology testing in some healthcare settings and that negative serology does not always indicate lack of prior HSV exposure. Whilst recommendations were made regarding screening for infective triggers in EM, no statements pertaining to possible triggers of SJS‐TEN were made in this study. Drugs are a more frequent cause of SJS‐TEN, and formal guidance regarding investigation and management for drug‐triggers is already available.^11^

A major limitation of this study is potential bias due to the predominance of both the members of the steering committee – 80% (4/5) – and of the expert panel – 47% (16/34) being from the UK. This suggests that both the statements and the consensus reached may be more reflective of UK practice than other healthcare systems and limits the generalizability of the recommendations. The available evidence base focused on oral care for this group of diseases is limited; in particular, there is a paucity of randomized trials, and in the available clinical trials, there is marked heterogeneity of outcomes and outcome measurement instruments.^17^ This means that many of the statements are primarily reflective of the clinical experience and expertise from the UK‐predominant members of the steering committee. Whilst this may be perceived as a weakness, it emphasizes the importance of a structured expert consensus as a tool for clinical decision‐making, which this study provides. Furthermore, in view of the high level of consensus achieved (93%) for the statements proposed by experts in the field, these statements appear to reflect expert opinion within the specialities involved in oral care, across differing healthcare systems.

For the first time we have provided consensus recommendations on the management of oral involvement in AIBD, SJS‐TEN and EM, which can be used in routine practice (Table 1). It is acknowledged that the statements may require adaptation by clinicians in their individual environments to deliver patient‐centred care, which reflects the specific patient needs within individual populations, the constraints and requirements of differing healthcare systems, and the specific expertise of the available multidisciplinary team.

**TABLE 1:** Summary of recommendations from the Delphi exercise. Scope of practice for clinicians will vary according to healthcare system and geographical location. Please see full text for further details.

General Recommendations	Topical therapies	Systemic therapies	Inpatient & Supportive Care
**Oral hygiene:**	**Topical corticosteroids:**	**Escalation:**	**Lip care:**
Daily antiseptic mouthwash SLS‐free toothpaste Soft toothbrush	Moderate‐potency mouthwash Super‐potent ointments for severe disease or gingival disease	Consider systemic therapy for moderate‐to‐severe oral lesions	Apply yellow soft paraffin Soak and debride lips with damp gauze
**Dental care:**	**External lip involvement:**	**Combination:**	**Oral hygiene:**
Regular dental and periodontal care Defer complex treatment until disease stabilized	Moderate‐potency corticosteroid ointment or tacrolimus ointment	Continue topical therapies and hygiene measures alongside systemic therapy	Supervised or assisted oral hygiene Twice‐daily antiseptic mouthwash
**Disease severity scoring:**	**Recalcitrant lesions:**		**Pain management:**
Validated scoring systems to guide treatment	Consider tacrolimus ointment or intralesional corticosteroid injection		Regular topical anaesthesia

EM / SJS‐TEN Specific
**Nutritional Support:** Initiate enteral feeding for extensive involvement
**Topical therapies:** Consider tranexamic acid‐soaked gauze for lip bleeding
**Sequelae of SJS‐TEN:** Sweep oral mucosa several times daily to prevent adhesions
**Acute EM:** Consider short courses of systemic corticosteroids
**Triggers for EM:** Consider HSV trigger in recurrent EM and prescribe antiviral prophylaxis Test for/treat *M. pneumoniae* where respiratory symptoms are present

## CONCLUSIONS

This DELPHI sets out a broad framework for the management of oral involvement in AIBD, SJS‐TEN and EM for both acute and long‐term management. These statements provide a clear outline for clinicians regarding appropriate topical, systemic and supportive management in this group of conditions and may therefore help to standardize management in future multicentre studies.

## AUTHOR CONTRIBUTIONS

Shalini Nayee: Data curation, Project administration, Writing – original draft. Molly Harte: Data curation, Project administration, Writing – original draft. Saskia‐Ingen‐Housz‐Oro: Conceptualization, Supervision, Funding acquisition, Writing – original draft, Writing – review & editing. Milad Ameri: Formal analysis, Writing – original draft. Pascal Joly: Conceptualization, Writing – review & editing. Sarah Walsh: Conceptualization, Supervision, Writing – original draft, Writing – review & editing. Jane Setterfield: Conceptualization, Supervision, Writing – original draft, Writing – review & editing. The Delphi expert panel: Writing – review & editing.

## FUNDING INFORMATION

Funding by ERN‐skin.

## CONFLICT OF INTEREST STATEMENT

Sarah Walsh declares her position as Associate Editor for JEADV. Marie‐Charlotte Brueggen declares contracts with GKS, CK‐CARE, LEO Foundation and LEO Pharma, USZ Innovasionspool, Astra Zaneca and the Swiss National Science Foundation. She has received consulting fees and/or payment from Sanofi, LEO Pharma, Alimirall, GSK and AbbVie. Serling Chua declares her position as chair for the British Association of Dermatologists' therapy and guidelines subcommittee and member of the guideline development group of the British Association of Dermatologists' clinical guidelines on Stevens‐Johnson Syndrome and Toxic Epidermal Necrolysis in adults. Donna Culton declares receiving an educational grant from Janssen. She declares status as a primary investigator with institutional payments from Cabaletta, Incyte, Regeneron, Lilly, Priovant and Pfizer. She declares payment from UpToDate and consulting fees from Sitala, Argenx, Icell, Sanofi, Regeneron, Novartis and Priovant. She declares positions on advisory boards for Argenx, Sanofi, Regeneron and Novartis. She declares unpaid positions on the international pemphigus and pemphigoid foundation, medical advisory board, US immunobullous consortium and AAD advisory board. She declares a family member with shares in Incyte.

Sabine Jurge declares employer payments for attendance at oral medicine conferences and for lectures of undergraduate and postgraduate students, as well as dentists and doctors. Dedee Murrell discloses position as an investigator for AbbVie, Amryt, Arena, ArgenX, Attovia, BMS, Castlecreek, Dermira, Evelo, Fujisawa, Galderma, Incyte, Innovaderm, Janssen, Kinseka, Leo, Lilly, Novartis, Pfizer, Principiabio, Regeneron, Roche, Sanofi, Shire, TWI, UCB and as an advisor for AbbVie, Almirall, Amryt, ArgenX, Castlecreek, Galderma, Innovaderm, Janssen, Krystal biotech, Lilly, Novartis, Nurix, Ono, Paradigm, Pfizer, Prinicpiabio, RAPT, Regeneron, RHeacell, Roche, Sanofi, Shire, TWI. She declares payment as a speaker for AbbVie, Amryt, ArgenX, Castlecreek, Galderma, Principiabio, Regeneron, Sanofi, Shire and TWI. Enno Schmidt declares institutional grants/contracts with Frensius Medical Care, Novartis, Incyte, Biotest, ArgenX, Dompe, Admirx InstitutionCSL, AstraZeneca, Bayer, Pharmaxis, Alpine Immune, Euroimmun, Sanofi. He declares personal consulting fees from Chugia, ArgenX, Janssen, BioCrust, Sanofi, Ono and AstraZenica and personal speaker fees from Sanofi, Almirall, AbbVie and Frensius Medical Care. He declares status as chair for the EADV AIBD task force and as a scientific coordinator for EADV/EDF guidelines. He declares financial support for attending meetings from Sanofi and Almirall. He declares advisory board positions with ArgenX, Sanofi and Chugai. He has patents planned/pending with Dompe Institution and Euroimmun. Tom Sollecito declares honoraria for lectures, presentations and manuscript writing. Shalini Nayee, Molly Harte, Saskia Ingen‐Houz‐Oro, Milad Ameri, Pascal Joly, Jane Setterfield, Michael Ardern‐Jones, Vlaho Brailo, Christopher Bunker, Barbara Carey, Marco Carrozzo, Daniel Creamer, Konstantina Delli, Marcio Diniz‐Freitas, Roni Dodiuk‐Gad, Sophie‐Miriam Dridi, Anne‐Laure Ejeil, Lars French, Krisztian Gaspar, Jon Higham, Barbara Hovarth, Esther Hullah, Roddy McMillan, Helen McParland, Martyn Ormond, Ana Poveda‐Gallego, Mahtab Samimi, Martina Shephard, Jennifer Taylor, Soner Uzun, Skaidra Valiukeviciene and Amanda Willis have no conflict of interest to disclose.

## ETHICAL APPROVAL

This study was not subject to ethical approval as it did not involve patient data or samples.

## ETHICS STATEMENT

The patients who provided images for this manuscript have given written informed consent to publication of their clinical images.

## Supporting information


**Table S1.** Country of practice of Delphi participants.

**Table S2.** Items of the Delphi exercise for the management of oral involvement in autoimmune bullous diseases, epidermal necrolysis and erythema multiforme. The panel were asked to score their level of agreement with each statement using a scale from 1 (strongly disagree) to 9 (strongly agree). DI (disagreement index) value greater than 1 indicates a lack of consensus; below 1 indicates a consensus. Statements with good agreement are considered ‘appropriate’.

Data S1.

## Data Availability

Data sharing not applicable to this article as no datasets were generated or analysed during the current study.
