# Supplementary material for: Expert consensus on oral management in autoimmune bullous diseases, erythema multiforme and SJS/TEN
Source: J Eur Acad Dermatol Venereol. 2026 Jul 22;40(11):1756–64. doi: 10.1111/jdv.70578 (PMC13695787; doi:10.1111/jdv.70578)
Supplement: Supplementary file 1 [file JDV-40-1756-s001.docx]

**Supplementary Data Table 1**

Items of the Delphi exercise for the management of oral involvement in autoimmune bullous diseases, epidermal necrolysis, and erythema multiforme. The panel were asked to score their level of agreement with each statement using a scale from 1 (strongly disagree) to 9 (strongly agree). *DI (disagreement index) value greater than 1 indicates a lack of consensus; below 1 indicates a consensus.* Statements with good agreement are considered “appropriate”.

**Items the panel agreed were “appropriate”.**

| **Items** | **DI*** | **Median** |
| --- | --- | --- |
| **I.** **General recommendations, oral hygiene and dental care** | | |
| Oral involvement in multi-site AIBD / EM / SJS-TEN SHOULD, if possible, involve collaboration between the dermatologist and an oral medicine specialist / stomatologist with specific expertise | 0 | 9 |
| Patients with isolated oral involvement of AIBD or EM MAY be managed by an oral medicine specialist or stomatologist with specific expertise alone | 0.599 | 8.5 |
| Attending clinicians SHOULD be aware that oral mucosal lesions in AIBD / EM / SJS-TEN take much longer to respond to topical and systemic therapies than cutaneous sites of disease | 0.056 | 9 |
| All patients with oral mucosal involvement in AIBD SHOULD be advised of the benefits of smoking cessation for their general oral health | 0 | 9 |
| Patients with oral involvement of AIBD / EM / SJS-TEN SHOULD receive advice from a dietician when oral intake is affected | 0.132 | 9 |
| Patients with oral mucosal involvement in AIBD SHOULD be advised that poor oral hygiene may be associated with a deterioration in oral lesions | 0 | 9 |
| All patients with oral mucosal involvement of AIBD SHOULD receive tailored oral hygiene instruction from a dental care professional at diagnosis | 0.292 | 9 |
| All patients with oral mucosal involvement of AIBD SHOULD receive careful periodontal care / dental hygiene advice every 3-6 months | 0.292 | 7.5 |
| Local anaesthetic SHOULD be considered for all patients with oral mucosal involvement of AIBDs undergoing periodontal therapy | 0.1 | 9 |
| All patients with oral mucosal involvement in AIBD SHOULD be advised to use sodium-lauryl-sulfate (SLS)-free toothpastes, which are less irritant | 0.292 | 9 |
| Patients with oral mucosal involvement in AIBD / EM / SJS-TEN COULD be advised to use a soft toothbrush | 0.056 | 9 |
| All patients with oral mucosal involvement in AIBD or EM with compromised oral hygiene SHOULD be advised to use an antiseptic mouthwash once daily, e.g., chlorhexidine 0.2% mouthwash as a rinse and spit preparation | 0.492 | 8 |
| Patients with oral mucosal involvement of AIBD SHOULD be advised that dental implants SHOULD NOT be performed until their disease is controlled | 0.132 | 9 |
| All patients with oral mucosal involvement of AIBD SHOULD be advised that their oral hygiene must be optimised before placement of dental implants | 0 | 9 |
| Bacterial, viral or fungal superinfection SHOULD be considered, investigated and treated where there is clinical concern | 0 | 9 |
| **II.** **Disease severity scoring** | | |
| A validated scoring system, depending on the disease (pemphigus vulgaris, mucous membrane pemphigoid), SHOULD be used to assess the severity of the oral disease involvement | 0.132 | 9 |
| A validated scoring system SHOULD guide the need for topical or systemic treatment of the underlying disease | 0.292 | 9 |
| A validated scoring system SHOULD be used to assess treatment response | 0.292 | 9 |
| **III.** **Topical therapies for active oral involvement in AIBD** | | |
| First-line topical therapy for mild oral mucosal involvement in AIBD SHOULD comprise a moderate potency corticosteroid mouthwash preparation, e.g., betamethasone or prednisolone soluble tablets as mouthwash | 0.2 | 9 |
| First-line topical therapy for moderate-severe oral mucosal involvement of AIBD, MAY comprise the use of both corticosteroid mouthwashes and ointments, in addition to systemic treatment e.g., fluticasone mouthwash and clobetasol ointment | 0.132 | 9 |
| First-line topical therapy for gingival predominant oral mucosal involvement in AIBD SHOULD comprise a super-potent topical corticosteroid ointment used once daily, e.g., clobetasol propionate ointment | 0.387 | 7 |
| For gingival predominant oral mucosal involvement in AIBD, the use of custom-made soft dental trays for use with a super-potent corticosteroid ointment MAY be considered, e.g. clobetasol propionate ointment | 0.376 | 8 |
| Super-potent corticosteroid ointments MAY be used as a maintenance therapy twice weekly for oral mucosal involvement in AIBD | 0.492 | 8 |
| Where high- or super-potency topical corticosteroid preparations are used, combined therapy with an antifungal agent SHOULD be considered to minimise the risk of recurrent candidosis | 0.132 | 9 |
| Patients SHOULD be advised to use their corticosteroid mouthwash at separate time to their antiseptic mouthwash | 0.132 | 9 |
| Super-potent corticosteroid ointments SHOULD NOT routinely be used for external lip involvement in AIBD patients in an outpatient setting | 0.148 | 9 |
| Where external lip involvement is present in AIBD, topical moderate potency corticosteroid ointment COULD be considered for once-twice daily application until lesions have healed | 0.292 | 8 |
| Where external lip involvement is present in AIBD, topical tacrolimus 0.1% ointment MAY be considered for twice daily application until healing is achieved | 0.132 | 9 |
| Topical tacrolimus 0.1% ointment MAY be considered for patients with oral mucosal involvement of AIBDs where topical corticosteroids are contraindicated or not available | 0.2 | 9 |
| Topical tacrolimus 0.1% MAY be considered as an adjunct to topical corticosteroids where oral mucosal lesions in AIBDs are recalcitrant | 0.132 | 9 |
| Intralesional injections of moderate potency corticosteroid, e.g., triamcinolone acetonide, COULD be considered for localised, recalcitrant oral mucosal ulceration in AIBD | 0.132 | 9 |
| **IV.** **Systemic therapies for active oral involvement in AIBD** | | |
| Systemic therapy MUST be considered in patients with moderate to severe oral mucosal lesions of any AIBD | 0 | 9 |
| The choice of systemic therapy MUST be guided by the AIBD diagnosis and sites of disease involvement | 0 | 9 |
| Systemic management for pemphigus vulgaris MUST be guided by the most up to date expert group guidelines | 0 | 9 |
| Systemic management for mucous membrane pemphigoid MUST be guided by the most up to date expert group guidelines | 0 | 9 |
| Topical therapies and oral hygiene recommendations for oral mucosal involvement in AIBD as described above SHOULD continue to be used as adjunct to systemic therapies | 0 | 9 |
| Clinicians SHOULD search for the best minimal effective dose of the systemic therapy to limit side effects | 0 | 9 |
| **V.** **Additional considerations for inpatient care of AIBD, EM, SJS-TEN** | | |
| Patients admitted with oral mucosal involvement in AIBD / EM / SJS-TEN SHOULD be assisted with oral hygiene by an appropriate allied health care professional such as a nurse | 0.056 | 9 |
| Patients admitted with oral mucosal involvement in AIBD / EM / SJS-TEN SHOULD use an antiseptic mouthwash e.g., chlorhexidine 0.2% mouthwash twice daily | 0.292 | 9 |
| Where patients admitted with oral mucosal involvement of AIBD / EM / SJS-TEN are unable to rinse and expectorate, antiseptic mouthwashes SHOULD be applied using a sponge | 0.292 | 8 |
| Patients admitted with oral mucosal involvement in AIBD / EM / SJS-TEN SHOULD be prescribed regular topical analgesia, e.g., oxetacaine plus antacid oral suspension, or lidocaine ointment or lidocaine spray or lidocaine naphazoline mixed in the mouthwashes | 0.132 | 8.5 |
| Patients admitted with external lip involvement of AIBD / EM / SJS-TEN SHOULD receive regular lip care including application of yellow soft paraffin | 0 | 9 |
| Patients admitted with external lip involvement in AIBD / EM / SJS-TEN SHOULD have the lips soaked and debrided with damp gauze to facilitate placement of ointments | 0.198 | 8 |
| **VI.** **Specific considerations for EM, SJS-TEN** | | |
| Patients with EM with severe oral involvement should be considered for hospital admission | 0.254 | 8 |
| Supportive care and systemic management for SJS-TEN SHOULD be guided by the most up to date guidelines | 0 | 9 |
| Patients admitted with extensive oral mucosal involvement in EM / SJS-TEN SHOULD receive enteral feeding (except where there is proven oesophageal involvement) | 0.492 | 7 |
| Mouth sponges SHOULD be used several times daily to sweep the oral mucosa to reduce the formation of mucosal adhesions and fibrosis associated with oral mucosal involvement in EM / SJS-TEN | 0.292 | 8.5 |
| Topical corticosteroids MAY be used in oral care of EM / SJS-TEN | 0.132 | 9 |
| First-line topical therapy for oral mucosal involvement of EM / SJS-TEN in an inpatient setting COULD comprise high potency topical corticosteroid mouthwash, e.g., fluticasone propionate | 0.132 | 9 |
| First-line topical therapy for lip or oral mucosal involvement in EM in an inpatient setting COULD comprise super-potent topical corticosteroid ointment, e.g., clobetasol propionate | 0.132 | 9 |
| Gauze soaked in tranexamic acid MAY be used to manage bleeding from the lips in patients admitted for EM / SJS-TEN | 0.292 | 8 |
| In acute EM, a short course of systemic corticosteroids MAY be given at time of diagnosis | 0.055 | 9 |
| In recurrent EM, herpes simplex MUST be considered as a potential trigger | 0 | 9 |
| In HSV-related recurrent EM with 2 or more episodes in one year, 6-12 months of prophylactic acyclovir/valaciclovir SHOULD be prescribed | 0 | 9 |
| Mycoplasma pneumoniae SHOULD be considered as a trigger where there is a first presentation of EM with associated pulmonary symptoms | 0 | 9 |
| M. pneumoniae investigations SHOULD include a nasopharyngeal PCR and a serology | 0.132 | 9 |
| Appropriate antimicrobials e.g. macrolides, SHOULD be prescribed in any case of EM with M. pneumoniae as suspected or proven trigger | 0 | 9 |
| After the acute phase of EM / SJS-TEN, regular visits SHOULD check the complete healing of the oral mucosa | 0 | 9 |

**Items the panel agreed were “uncertain”**

| **Items** | **DI** | **Median** |
| --- | --- | --- |
| **I.** **General recommendations, oral hygiene and dental care** | | |
| All patients with oral mucosal involvement in AIBD, EM, SJS-TEN with compromised oral hygiene SHOULD be advised to use hydrogen peroxide 1.5% mouthwash daily for short term use | 0.851 | 5 |
| **III.** **Topical therapies for active oral involvement in AIBD** | | |
| Super-potent corticosteroid ointments SHOULD NOT routinely be used daily for more than 14 days for oral mucosal involvement in AIBD without careful review | 0.652 | 6.5 |
| **V.** **Additional considerations for inpatient care of AIBD, EM, SJS-TEN** | | |
| Patients admitted with oral mucosal involvement in AIBD / EM / SJS-TEN SHOULD use an antiseptic mouthwash, e.g., chlorhexidine 0.2% mouthwash, 4 to 6 times daily | 0.806 | 3 |
| **VI.** **Specific considerations for Erythema Multiforme (EM), Stevens-Johnson Syndrome and Toxic Epidermal Necrolysis (SJS-TEN)** | | |
| When oral lesions are present in recurrent EM, herpes simplex virus serology results SHOULD be used to guide prophylactic treatment | 0.652 | 6 |
