# Supplementary material for: Expert consensus on oral management in autoimmune bullous diseases, erythema multiforme and SJS/TEN
Source: J Eur Acad Dermatol Venereol. 2026 Jul 22;40(11):1756–64. doi: 10.1111/jdv.70578 (PMC13695787; doi:10.1111/jdv.70578)
Supplement: Supplementary file 2 [file JDV-40-1756-s002.docx]

And the Delphi expert panel: Michael Ardern-Jones^10^, Vlaho Brailo^11^, Marie-Charlotte Brueggen^12,13,14^, Christopher Bunker^15^, Barbara Carey^16^, Marco Carrozzo^17^, Ser-Ling Chua^18^, Daniel Creamer^6^, Donna Culton^19^, Konstantina Delli^20,21^, Marcio Diniz-Freitas^22,23^, Roni Dodiuk-Gad^24^, Sophie-Myriam Dridi^25^, Anne-Laure Ejeil^26,27,28^, Lars French^29^, Krisztian Gaspar^30,31^, Jon Higham^32^, Barbara Horvarth^33^, Esther Hullah^1^, Sabine Jurge^34^, Roddy McMillan^35^, Helen McParland^1^, Dedee Murrell^36^, Martyn Ormond^1^, Ana Poveda-Gallego^32^, Mahtab Samimi^37^, Enno Schmidt^38,39^, Martina Shephard^35^, Tom Sollecito^40^, Jennifer Taylor^41^, Sonur Uzun^42^, Skaidra Valiukeviciene^43^, Amanda Willis^44^

10. Department of Dermatology, University Hospitals Southampton NHS Foundation Trust, UK
11. University of Zagreb School of Dental Medicine, University Clinical Hospital Centre Zagreb, Croatia
12. Department of Dermatology, University Hospital of Zurich, Zurich, Switzerland
13. Faculty of Medicine, University of Zurich, Zurich, Switzerland
14. Department of Dermatology, Medical University of Vienna, Vienna, Austria
15. Department of Dermatology, University College London Hospitals, London, UK
16. Department of Head & Neck Surgical Oncology, Guy’s Hospital, London, UK
17. Department of Oral Medicine, School of Dental Sciences, University of Newcastle upon Tyne, UK
18. Department of Dermatology, University Hospitals Birmingham NHS Foundation Trust
19. Department of Dermatology, University of North Carolina, Chapel Hill, North Carolina, USA
20. Department of Oral and Maxillofacial Surgery, University of Groningen, University Medical Centre Groningen, Groningen, the Netherlands
21. Center for Dentistry and Oral Hygiene, University Medical Center Groningen and University of Groningen, Groningen, the Netherlands
22. Special Care Dentistry Unit, School of Medicine and Dentistry, University of Santiago de Compostela, Santiago de Compostela, Spain
23. Medical-Surgical Dentistry Research Group (OMEQUI), Health Research Institute of Santiago de Compostela (IDIS), University of Santiago de Compostela (USC), Santiago de Compostela, Spain
24. Department of Dermatology, Emek Medical Center, Afula, Israel
25. Fondation Université Côte d’Azur, Department of Periodontology, Nice, Provence-Alpes-Côte d'Azur, FR
26. Department of Odontology, Bretonneau Hospital AP-HP, Paris, France
27. Faculty of Odontology, Department of Oral Surgery, Paris Cité University, Montrouge, France
28. Inserm UMR1333 Oral Heath, Montrouge, France
29. Department of Dermatology and Allergy, University Hospital, LMU Munich, Munich, Germany
30. Division of Dermatological Allergology, Faculty of Medicine, University of Debrecen, Debrecen, Hungary
31. Department of Dermatology, Faculty of Medicine, University of Debrecen, Debrecen, Hungary
32. Department of Oral Medicine, Birmingham Dental Hospital and School of Dentistry, Birmingham, UK
33. Department of Dermatology University Medical Centre Groningen, University of Groningen, Groningen, The Netherlands
34. Department of Oral Medicine, Sheffield Teaching Hospitals NHS Foundation Trust and University of Sheffield
35. Department of Oral Medicine & Facial Pain, Royal National ENT and Eastman Dental Hospitals, University College Hospitals, University College London Hospitals NHS Foundation Trust, UK
36. Department of Dermatology, St George Hospital Clinical School, University of NSW, Sydney, Australia
37. Dermatology Department, University Hospital of Tours, France
38. Lübeck Institute of Experimental Dermatology, University of Lübeck, Lübeck, Germany
39. Department of Dermatology, Universitätsklinikum Schleswig-Holstein, Campus Lübeck, Lübeck, Germany
40. School of Dental Medicine, University of Pennsylvania, USA
41. Oral Medicine, University of Glasgow, Glasgow Dental Hospital and School, Glasgow, UK
42. Department of Dermatology, Faculty of Medicine, Akdeniz University, Antalya, Turkey
43. Department of Skin and Venereal Diseases, The Lithuanian University of Health Sciences, Kaunas, Lithuania
44. Department of Oral Medicine, Queens University Belfast, Belfast Health and Social Care Trust, Belfast, UK
