# Supplementary material for: Expert consensus on oral management in autoimmune bullous diseases, erythema multiforme and SJS/TEN
Source: J Eur Acad Dermatol Venereol. 2026 Jul 22;40(11):1756–64. doi: 10.1111/jdv.70578 (PMC13695787; doi:10.1111/jdv.70578)
Supplement: Supplementary file 3 [file JDV-40-1756-s003.docx]

**Supplementary Data Table 2**

Country of practice of Delphi participants

| **Country** | **Number of experts who participated** | **Number of experts who did not respond** |
| --- | --- | --- |
| UK | 16 | 14 |
| France | 4 | 8 |
| Germany | 2 |  |
| Netherlands | 2 |  |
| USA | 2 | 1 |
| Australia | 1 |  |
| Croatia | 1 |  |
| Hungary | 1 |  |
| Israel | 1 |  |
| Lithuania | 1 |  |
| Spain | 1 |  |
| Switzerland | 1 |  |
| Turkey | 1 | 1 |
| Portugal |  | 1 |
| Italy |  | 2 |
| Czech Republic |  | 1 |
| Belgium |  | 2 |
| Hungary |  | 1 |
| Total | 34 | 31 |
